# Correspondence

**Published:** 1869-01

**Authors:** M. McCarty


### Correspondence.

EDITORS REGISTER: In the September number of the REGISTER some one, under the *nom de plume* of "Emery," answers my communication in the July number. One point in my article he seems to doubt, another he attempts to controvert, while as to another he claims to give a simpler method. As to the first point, as he did not discuss it, no answer is required. But the next point needs some explanation from me. I readily admit that Prof. Taft said that arsenious acid produced death in the Dental pulp, by entering into the circulation, and in no other way. But in this I do not admit that he was supported by his associate professors, or by one of them, at least (I heard but one other speak on this subject.) If I recollect aright, Prof. Spaulding said that it was not known how arsenious acid produced death in the Dental pulp. For, that upon chemical analysis of a pulp that had been devitalized with arsenious acid, not a *trace* of the acid had been discovered. It was not my object, however, in writing my communication, to show how arsenious acid acted in devitalizing, but to show how to give it a fair chance to act effectually when we call it into use.

"Emery" objects to my practice, because it is too difficult and tedious, and produces too much pain, and proposes a "simpler method," that of putting the nerve paste upon the sharp point of an instrument and puncturing the pulp. Now, upon the trial of this experiment, he will find that the paste will, in many cases, be pushed up the blade of the instrument just as far as it enters the pulp, and will consequently be withdrawn with it; or that the orifice closing up, the paste will not come in contact with the capillaries, so that it can pass into the "circulation." Still there is another chance for a failure. The blood may wash the paste away from the orifice of the puncture.

Since my practice has been questioned, and I was not explicit about the procedure in my former article, I deem it advisable to give it in detail. Obtunde the sensibility of the pulp wherever it can be done with the narcotic spray, (always taking the precaution to protect the pulp with wax or cotton) or have a narcotic mixture of equal parts tinc. aconite, opium and belladonna, into which dip a pledget of cotton, and put in contact with the pulp a few minutes before cutting. Remove the cotton and cut off the surface of the pulp at one stroke; give time for the bleeding to subside, wash the cavity with tepid water, then wipe it dry. Spread the paste on a small pledget of cotton that has been previously moistened with carbolic acid; then place the pledget with the paste in contact with the cut surface of the pulp; on this pledget place a dry one, on the dry one another that has been dipped about half way its bulk in sandarach varnish. The pulp may be removed twelve or twenty-four hours afterwards.

"Emery" is undoubtedly very unhappy in his comparison when he says his plan is much more acceptable than mine. Just as the short, sharp work of the bullet would be more acceptable to the condemned than the lingering process of strangling with a rope, while, in reality, my process is the cutting off the head of the condemned (pulp) with an axe—his is the thrust of the dagger into the body of the condemned (pulp).

M. McCarty.

